# Preferred and Actual Relative Height among Homosexual Male Partners Vary with Preferred Dominance and Sex Role

**DOI:** 10.1371/journal.pone.0086534

**Published:** 2014-01-22

**Authors:** Jaroslava Varella Valentova, Gert Stulp, Vít Třebický, Jan Havlíček

**Affiliations:** 1 Center for Theoretical Study, Charles University in Prague and the Academy of Sciences of the Czech Republic, Prague, Czech Republic; 2 Department of Sociology, University of Groningen, Groningen, The Netherlands; 3 Department of Philosophy and History of Sciences, Faculty of Science, Charles University, Prague; 4 Department of Zoology, Faculty of Science, Charles University, Prague; University of Goettingen, Germany

## Abstract

Previous research has shown repeatedly that human stature influences mate preferences and mate choice in heterosexuals. In general, it has been shown that tall men and average height women are most preferred by the opposite sex, and that both sexes prefer to be in a relationship where the man is taller than the woman. However, little is known about such partner preferences in homosexual individuals. Based on an online survey of a large sample of non-heterosexual men (N = 541), we found that the majority of men prefer a partner slightly taller than themselves. However, these preferences were dependent on the participant’s own height, such that taller men preferred shorter partners, whereas shorter men preferred taller partners. We also examined whether height preferences predicted the preference for dominance and the adoption of particular sexual roles within a couple. Although a large proportion of men preferred to be in an egalitarian relationship with respect to preferred dominance (although not with respect to preferred sexual role), men that preferred a more dominant and more “active” sexual role preferred shorter partners, whereas those that preferred a more submissive and more “passive” sexual role preferred taller partners. Our results indicate that preferences for relative height in homosexual men are modulated by own height, preferred dominance and sex role, and do not simply resemble those of heterosexual women or men.

## Introduction

Human mate preferences and mate choice are known to be substantially influenced by physical characteristics of potential or actual mates [1], [2]. Previous research has repeatedly shown that sexually dimorphic traits positively affect mate choice criteria [3]. Human stature seems to be one such indicator: on average, men are taller than women [4], and height plays an important role in both mate preferences (reviewed in Courtiol et al. 2010 [5]) and choice [6]–[8]. In general, these studies reveal that, on average, tall men and average height women are most preferred by the opposite sex, and that both sexes prefer to be in a relationship where the woman is shorter than the man [9]–[12]; this is tempered, however, by recent evidence suggesting that the latter preference is stronger in women than in men [8], [13]. It should also be noted that these height preferences seem restricted to Western populations [14], [15].

As women place more value on their partner’s height than men do, it follows that height is more important for male than for female physical attractiveness [11], [13]. There is some evidence to suggest that the increased attractiveness of taller men extends from the laboratory into more naturalistic settings, as taller men are more successful during speed-dating [7], [8], have partners who are judged as more attractive [16], and report a higher number of sexual partners [17]. Some studies have even reported that taller men have higher reproductive success [18], [19], although a recent, comprehensive review suggests that, among Western populations, it is men of average height that produce the most offspring [20].

A potential evolutionary rationale for why women prefer taller men is that height acts as cue of male mate quality. Indeed, it has been shown that, on average, taller men are healthier [21]–[25] and live longer [26] than shorter men. Female preferences for male height may thus be interpreted as a preference for health and longevity in a mate. The fact that there are limits to female preferences for height adds circumstantial support to this argument: extremely tall men are considered less attractive as mates, and such men face a higher risk of cancer [27], and may display disorders such as pituitary gigantism and Marfan’s syndrome [28].

Height may also serve as a cue to male dominance (for review, see Buunk et al. 2008 [29]). Indeed, it has been shown that height is positively correlated with men’s physical strength [30], physical aggression [31], fighting abilities [32], striking force [33], as well as aspects of their social status [34], including income [35]. Moreover, people stereotypically judge tall men as more dominant and assertive [36]. From an evolutionary perspective, these findings suggest that height may serve as an indicator of competitive ability against rival males [28]. Thus, in addition to potential health and longevity benefits, women may prefer taller men because they are more likely to be dominant and hold higher social status.

Although, on average, women prefer taller over shorter men, while men prefer women of average height, [5], there are also systematic inter-individual differences in height preferences. That is, preferences for partner height are modulated by an individual’s own height: both taller men and women, for instance, prefer taller partners compared to shorter men and women [5]. Furthermore, taller men and shorter women tend to prefer larger partner height differences [10], [37] than those who are shorter, which is hypothesised to increase the pool of potential partners available to such individuals [10]. Such self-similarity preferences are also observed in actual pair formation: positive assortative mating with respect to height is a widespread phenomenon [38]–[40]. Finally, it has been shown that both men and women avoid extreme height differences in their partners: women prefer men not too tall compared to their own height, and men prefer women not too short [10], [13], [37]. This latter preference may also be adaptive, as women in couples with a larger than average height difference experience a higher risk of birth complications [41].

### The Current Study: Partner Preferences in Homosexual Men

Although heterosexual preferences and choice for partner height have received considerable attention, little is known about these among homosexual individuals. Previous studies report that homosexual men show a male-typical mating psychology, including an interest in casual sex and sexually explicit visual material [42], as well as showing male-typical mate retention behavior [43]. Similar to heterosexual men, homosexual men value physical attractiveness in their potential partners more than heterosexual women [44], and they prefer potential partners who are younger than themselves [45], [46]. Thus, it seems that the effect of gender on variation in partner preference is stronger than the effect of sexual orientation [47]. Having said this, homosexual men also prefer men who are described as typically masculine [48]; in particular, they prefer masculine male voices [49] and faces [50]. There are, however, striking individual differences in preferences for facial masculinity in homosexual men: single homosexual men prefer more masculine male faces than those in a relationship [49], and homosexual men who report higher levels of sexual desire also prefer more masculine male faces [51] (but see [49]).

Preferences for sex-typical traits (i.e., masculinity) in homosexual men are also influenced by their preferred role during sexual intercourse (i.e., adopting the ‘penetrating’ versus ‘penetrated’ role, or ‘top’ and ‘bottom’) – ‘top’ homosexual men prefer more feminine male faces, while ‘bottoms’ prefer more masculine male faces [52]. Based on a North American and Latino American sample, it has been suggested that anal sex between homosexual men not only represents activity leading to sexual pleasure, but is also connected to other personality, or interpersonal factors, such as masculinity and sexual power - the same being true for heterosexual individuals [53], [54]. Specifically, it has been shown that men who exhibit a passive role in sexual intercourse are perceived as more feminine, whereas the opposite is true of men exhibiting the active role. Furthermore, it has been shown that, on average, ‘tops’ report being more dominant or sexually aggressive, while ‘bottoms’ report being more submissive in sexual activities [53]. This behavior is also strongly connected to other active/passive or dominant/submissive sexual activities, such as fellatio, verbal abuse, and fisting [53]. Data from a Chinese sample has also shown that ‘top’ and ‘bottom’ homosexual men differ in personality characteristics, with ‘tops’ scoring higher on instrumentality and masculinity, while ‘bottoms’ score higher on expressiveness [55].

The main aim of the current study was to explore height preferences and actual partner height characteristics of homosexual men. Based on previous studies of heterosexual preferences, we predicted that, on average, taller men would be preferred as partners, but that relative height preferences would be modulated by an individual’s own stature. We also examined whether preferences were influenced by the preferred role adopted during sexual intercourse and the preferred dominance role in a relationship. In particular, we examined the influence of preferred and actual sexual roles (i.e., passive, active or switching, or ‘bottom’, ‘top’, or a ‘versatile’ role during sexual intercourse) on relative height preferences. Given the findings on preferences for facial masculinity [50], and the fact that human stature is a sexually dimorphic trait, we predicted that homosexual men who preferred to be in the ‘top’ position during sexual intercourse would also prefer relatively shorter partners, whereas those that preferred the ‘bottom’ position would prefer taller partners.

Finally, we investigated whether an individual’s own height, and the relative height of their preferred and actual partners was associated with preferred dominance status within a couple (i.e., the degree to which an individual wished to behave in a dominant or submissive way toward a partner). Given that height has been shown to relate strongly to dominance cues, we predicted this would be related to the dominance relationship between partners, with those expressing a preference to be more dominant preferring shorter partners relative to those who prefer to be submissive. In general, according to interpersonal theory, the dominance dimension is one of the two primary dimensions of interpersonal behavior (in addition to the affiliation dimension) [56], [57]. Complementarity of dominant and subordinate behavior also serves to regulate aggression and conflict and facilitates cohesion in social group encounters including dyads [58]. Since male body height is perceived as a cue to male dominance, we suggest that preferred height would be positively associated with preferred (and actual) dominance towards a potential and/or an actual partner. More precisely, we hypothesized that men who prefer to be rather dominant towards a potential partner will prefer shorter partners, while men preferring rather a submissive role in their relationships will show preferences for taller men, and a similar pattern was also expected to appear in actual same-sex couples.

## Materials and Methods

### 2.1 Participants

The sample consisted of 541 non-heterosexual male participants (M age = 26.37 years, SD = 6.43) who were recruited as a part of a larger study of male physical attractiveness. All participants were recruited via snowball sampling through the use of mailing-lists obtained for our previous studies, and through advertisements on Facebook. All participants were of Czech origin. Data were collected through an online questionnaire using Qualtrics (Qualtrics Inc., 2009). At the start of the survey, the participants gave their informed consent via an online form. This required a mouse-click to confirm their willingness to take part, and enabled them to proceed to the survey. Participants were presented with a series of questionnaires aimed at gathering demographic data, participants’ sexual history, self-evaluated attractiveness, and ratings of male physical attractiveness. Only measures relevant to this study are reported below. The study was approved by the Institutional Review Board on Human Subjects of the Faculty of Science, Charles University in Prague (nr. 2013/1).

### 2.2 Questionnaires

#### 2.2.1 Sexual orientation and relationship status

Sexual orientation was reported using a verbally anchored Kinsey scale, where 0 = exclusively heterosexual, 3 = bisexual, and 6 = exclusively homosexual. From the total sample, only men who reported they were either bisexual (N = 35; 6.5%), somewhat (N = 35; 6.5%), mostly (N = 147; 27.2%) and exclusively (N = 324; 59.9%) homosexual were included in the sample for further analyses. Results were very similar when we included only those men who reported they were ‘exclusively homosexual’, and when we excluded bisexual men (results not reported here); we thus present results for the entire non-heterosexual sample.

Participants were asked if they had a stable male sexual partner at the time of the study. In total, 44.3% (N = 230) of men reported having a stable male partner, 41.0% (N = 213) were single currently, but reported having a stable male partner previously, and 14.6% (N = 76) had never had a long-term male partner. There were missing values on relationship status for twenty-two men.

#### 2.2.2 Own height and relative stature preferences

**Figure 1:**
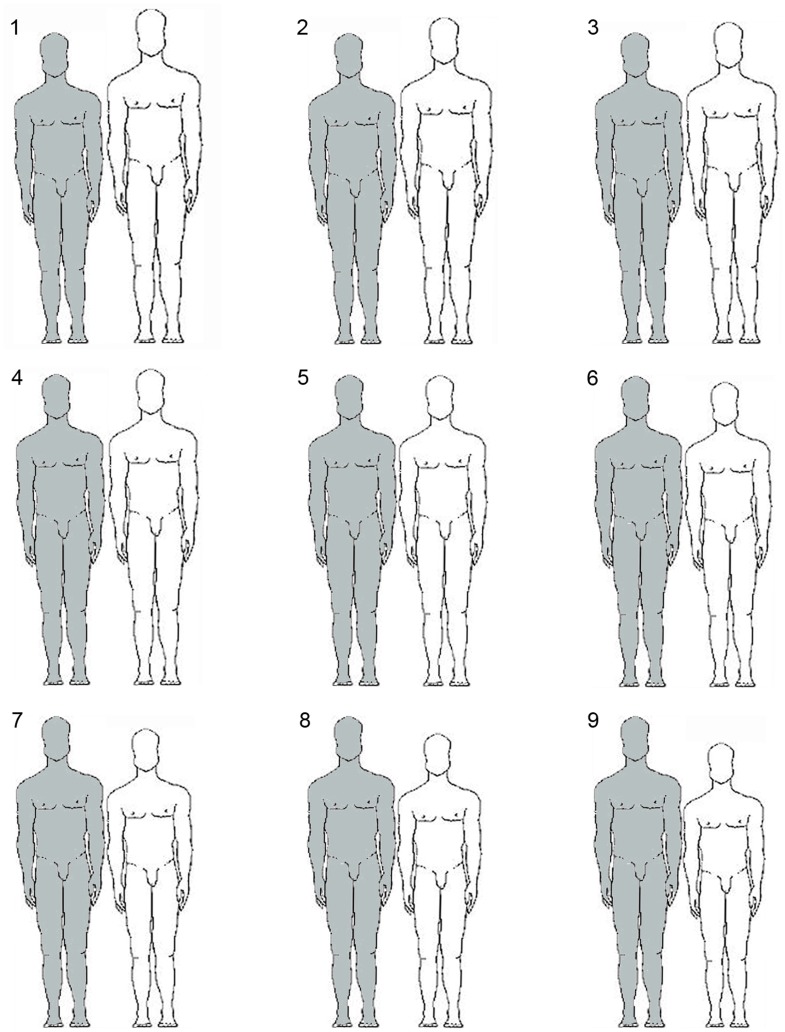
The stimuli used for assessing preferred and actual relative height among partners. The question asked: ‘Indicate your preferred/actual height of your partner (white figure) compared to your own height (grey figure) - individuals in couple 5 are of the same height’.

Each participant reported on his height (in cm). The mean height of the entire sample (N = 541) was 180.6 cm (SD = 6.75, range 158–202 cm). To assess preferences for stature differences between a participant and his ideal partner (i.e., preferences for relative height), and the actual stature differences of participants with a partner, we adapted the ‘Sexual dimorphism in stature’ scale [10], using only the male figures. Variation in height differences was set up in the following fashion: the size of the target figure representing the respondent was increased or decreased by 0.5, 1, 1.5, and 2 standard deviations of the average Czech male height (180 cm, SD = 6.5). The data on variation in male height was based on a representative sample of Czech adult men [59]. This resulted in 9 drawings of male couples that varied in their relative height, centred on a couple of equal height (see Figure 1). Participants were asked to select the drawing that depicted the preferred relative height difference between them and their ideal partner, and were subsequently asked to select the drawing that depicted the actual relative height difference between them and their actual partner. Participants that never had a same-sex relationship (N = 76), did not answer the above questions, resulting in a sample of (N = 465). Participants who were single at the time of the study, but reported having a stable relationship in the past, were asked to indicate the relative height difference between them and their most recent former partner.

#### 2.2.3 Sexual and dyadic dominance

Each participant (except those that reported that they never have had a same-sex partner) (N = 465) indicated his preference for a dominant or submissive role in a relationship (‘Please indicate whether, in your relationship, you would prefer to be dominant or submissive towards your partner’) using a 7-point scale, where 1 = very dominant and 7 = very submissive. Respondents who reported being in a stable relationship at the time of the study (N = 230) indicated their actual dominance. Finally, all respondents reported their usual role in sexual intercourse (‘Please indicate whether in sexual activities you are usually more active ( = ‘top’) or passive ( = ‘bottom’) using a 7-point scale where 1 = always active, 7 = always passive, and 4 means that ‘you don’t prefer a specific sexual role or you switch roles regularly’). Again, only respondents who reported being in a stable relationship at the time of the study (N = 230) indicated their current sexual role in the relationship.

### Statistical Analyses

We used Pearson correlations to examine the associations between self-reported height and preferred and actual relative height among partners (the data were normally distributed). We used Spearman’s rank correlations (r_s_) to test relationships between preferred and actual relative height among partners, and preferred and actual dominance, as one of the variables was measured on a 7-point scale, and the other on a 9-point scale. Given that we ran several correlation analyses, we have a greater probability of making a Type 1 error (i.e., rejecting H_0_ when H_0_ is true). After applying a Bonferroni correction, all reported findings remained significant, except one, which had an uncorrected p-value of.035 (which we also address by explicitly mentioning the low effect size). We report p-values without corrections. All analyses were performed using SPSS 17.0.

## Results

### 3.1 Preferred and Actual Relative Height Among Partners

**Figure 2:**
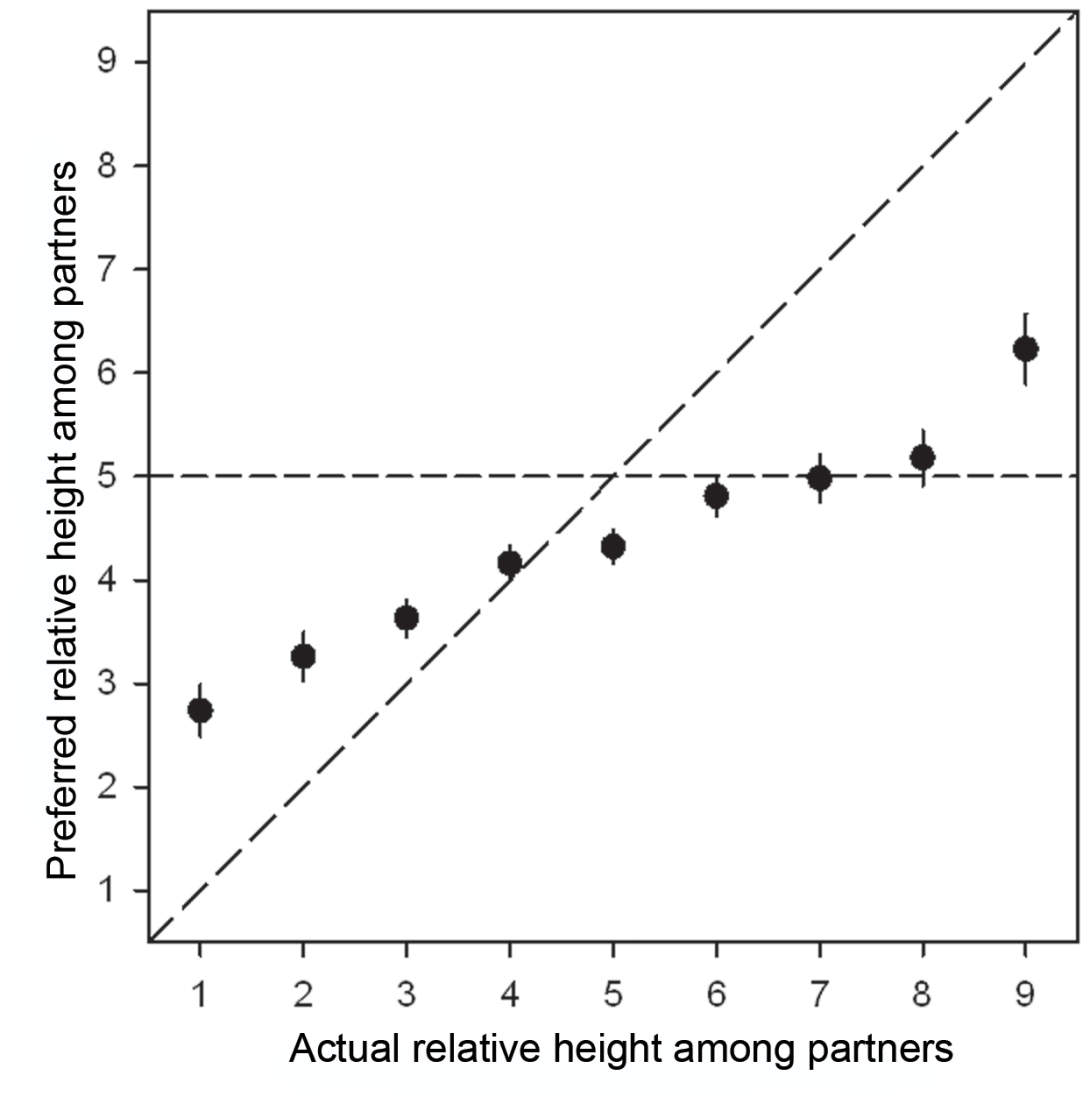
Preferred relative height among partners (mean ± SE) as a function of actual relative height among partners. The horizontal line reflects a preference for a partner of similar height. The diagonal line reflects (y = x).

As shown in Table 1 and Figure 2, 24.3% of men preferred a relationship in which partners were of similar height (i.e., they selected a drawing # 5 in Figure 1), 23.5% preferred a partner who was shorter than themselves (i.e., they selected drawings 6–9 in Figure 1), but the majority of men (52.3%) preferred a partner who was taller than themselves (i.e., they selected drawings 1–4 in Figure 1).

**Table 1:** Descriptive statistics for preferences and actual choice of the partner relative height. For both variables mean height (±SD) of the male participants is reported.

	Ideal partner			Actual partner	
Relative height among partners		N (%)	Mean height (±SD)	N (%)	Mean height (±SD)
1		34 (7.3%)	175.91 (±5.41)	46 (9.9%)	175.63 (±6.90)
2		41 (8.8%)	176.90 (±7.44)	46 (9.9%)	176.15 (±5.67)
3		77 (16.6%)	179.77 (±5.99)	43 (9.2%)	178.23 (±5.49)
4		91 (19.6%)	179.14 (±6.10)	69 (14.8%)	179.28 (±4.28)
5		113 (24.3%)	182.04 (±5.98)	82 (17.6%)	180.04 (±6.07)
6		65 (14.0%)	183.65 (±7.11)	64 (13.8%)	182.66 (±5.77)
7		29 (6.2%)	183.31 (±8.52)	51 (11.0%)	183.67 (±5.87)
8		10 (2.2%)	183.30 (±5.06)	38 (8.2%)	186.11 (±8.44)
9		5 (1.1%)	188.00 (±4.74)	26 (5.6%)	187.15 (±6.09)

Eighteen percent of men reported that they were in a relationship with a partner of similar height, approximately 39% were in a relationship with a relatively shorter partner and a substantial proportion of 44% of men were in a relationship with a taller partner (Table 2). Preferred relative height and actual relative height were correlated significantly (r = .487; p<.0001; N = 465), indicating that men in a relationship in which there was a large partner height difference also preferred a large partner height difference (Figure 2). Visual examination of Figure 2 shows that, on average, men with partners of the same height would have preferred to be shorter than their partners, whereas men who were either much taller or much shorter than their actual partners expressed a preference for a smaller height difference (Figure 2). This effect was more pronounced, however, among men who were much taller than their partners (Figure 2). A paired samples t-test indicated that, on average, men preferred smaller partner height differences than they actually experienced (mean difference: −.512 (±2.089); t = 5.284; df = 464; p<.0001; d = .245). In other words, most men would have preferred to be less tall or less short relative to their partner (Figure 2).

**Table 2:** Frequencies (%) of preferred dominance role, actual dominance role and actual sex role.

		Dominance role		Preferred sex role	
	Preferred		Actual		
Very dominant	8 (1.7%)		4 (1.7%)	Always active	14 (6.1%)
Dominant	47 (10.1%)		29 (12.6%)	Mostly active	25 (10.9%)
Slightly dominant	88 (18.9%)		43 (18.7%)	Sometimes active	22 (9.6%)
Neither dominant nor submissive	211 (45.4%)		101 (43.9%)	Neither active or passive	77 (33.5%)
Slightly submissive	90 (19.4%)		44 (19.1%)	Sometimes passive	30 (13.0%)
Submissive	18 (3.9%)		7 (3.0%)	Mostly passive	43 (18.7%)
Very submissive	3 (0.6%)		2 (0.9%)	Always passive	19 (8.3%)

### 3.2 The Association between Own Height and Relative Partner Height

**Figure 3:**
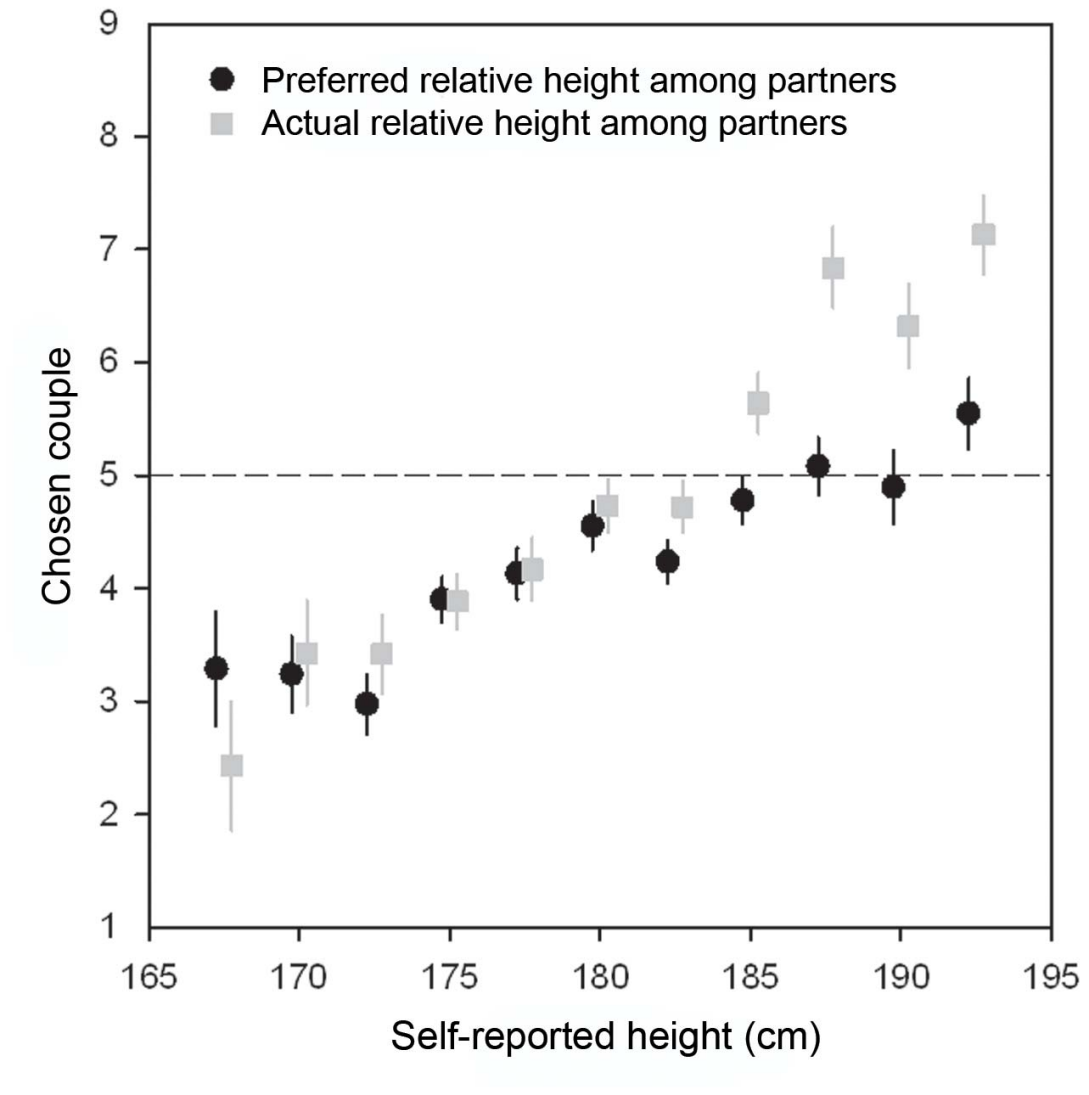
The relationship between own height and preferred and actual relative partner height (mean ± SE). Height was divided into 2.5(bins below 167.5 and above 192.5 were collapsed). The horizontal line reflects no partner height difference. Height correlated positively with both preferred and actual relative height among partners.

Reported height of the respondents was positively associated with ideal partner height (Table 2; r = .347; p<.0001; N = 465). Figure 3 shows that very tall men preferred to be slightly taller than their partner, whereas average height and short men preferred to be (slightly) shorter than their ideal partner. Respondent’s height was also correlated with actual relative height among partners (Table 2; r = .495; p<.0001; N = 465); tall men were, on average, much taller than their partners, whereas short men were, on average, much shorter than their partners (Figure 3).

Height correlated negatively with the difference between ideal and actual relative height among partners (r = −.247; p<.0001; N = 465). Most notably, taller men expressed a preference for a smaller height differences than they actually experienced (Figure 3).

### 3.3 The Association between Dominance, Sexual Role, and Relative Height Among Partners

**Figure 4:**
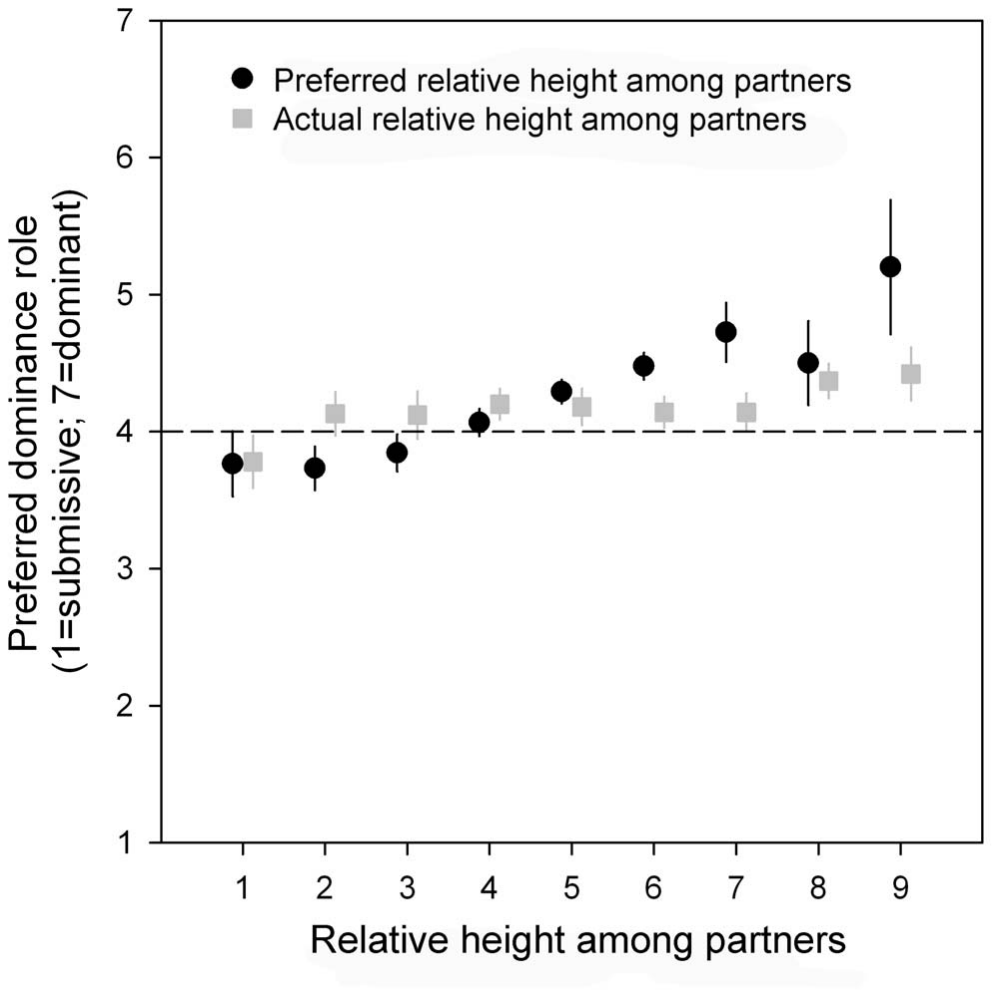
The correlation between preferred/actual relative height among partners and preferred dominance role (mean ± SE). The horizontal line reflects neither submissive nor dominant.

The largest proportion of men (45.4%) preferred to be in a relationship in which they were neither dominant nor submissive (Table 2). With respect to preferred relative height among partners, we found that preferred dominance role was positively associated with preferred relative height among partners (Figure 4; r_s_ = .303; p<.0001; N = 465), which means that men who preferred to be much taller than their partner also preferred to be much more dominant, whereas men who preferred to be much shorter than their partner preferred to be slightly submissive (Figure 4). Similar effects were found with respect to actual relative height among partners and preferred dominance role (although much weaker; Figure 4; r_s_ = .098; p = .035; N = 465). Preferred and actual dominance were strongly correlated (r_s_ = .616; p<.0001; N = 230). Self-reported height of the respondents was not correlated to either preferred dominance (r_s_ = .013; p = .784; N = 465), or actual dominance (r_s_ = .017; p = .803; N = 230), indicating that actual an individual’s height was not related to being or preferring to be dominant towards the partner.

**Figure 5:**
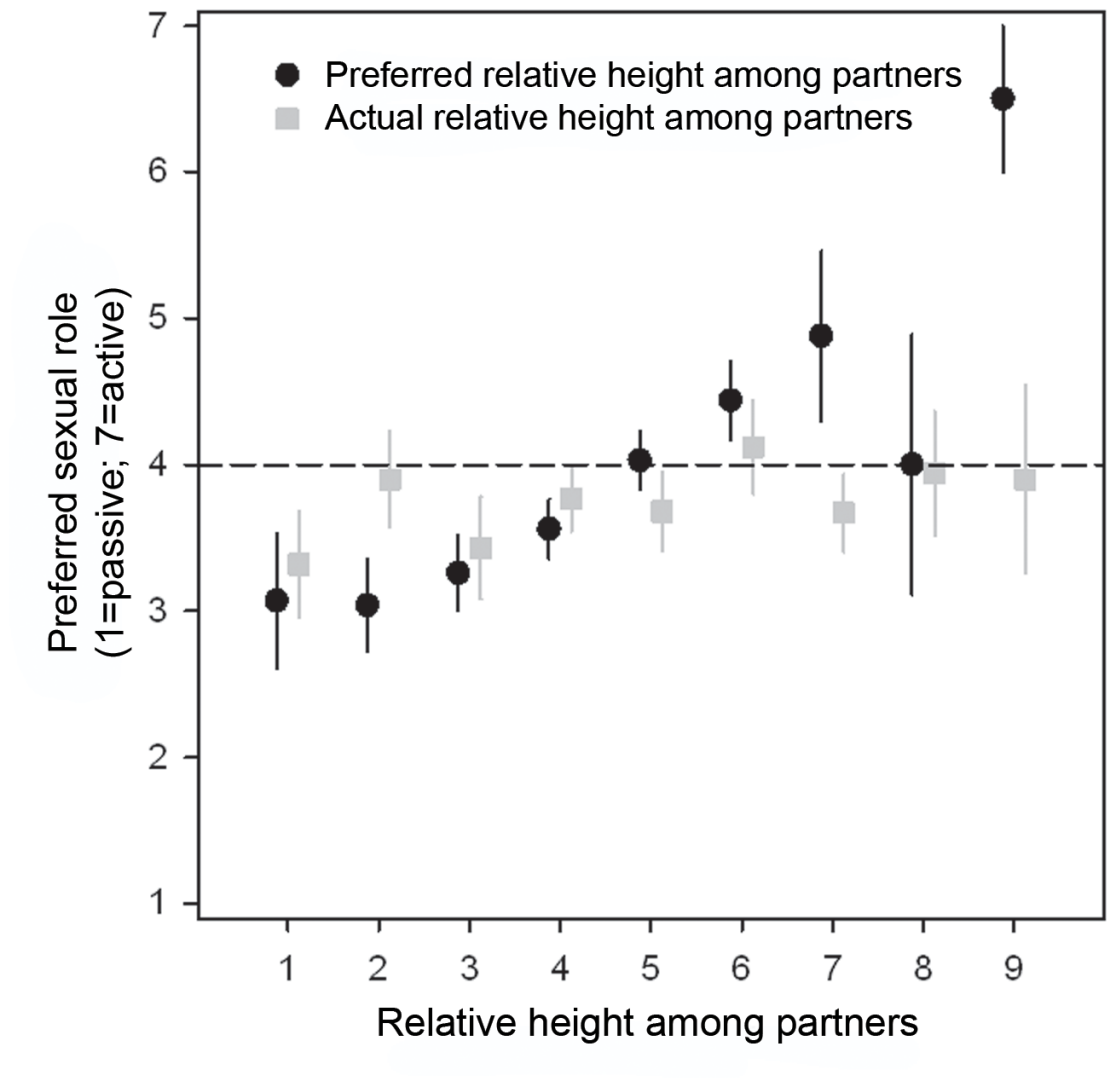
The correlation between preferred/actual relative height among partners and sexual role (mean ± SE). The horizontal line reflects neither passive nor active role during intercourse.

Preferred dominance also correlated positively with preference for a sexual role (r_s_ = .404; p<.0001; N = 230); men who preferred to be relatively dominant in their relationship also preferred to take the active role during sexual intercourse. A substantial proportion of men (33.5%) preferred being neither passive nor active during sexual activities, or they switched roles regularly (Table 2). Results for the association between relative height among partners and preferred sex role within a relationship were very similar to those concerning the preferred dominance role. Preferred relative height among partners was positively associated with preferred sex role (r_s_ = .320; p<.0001; N = 230; Figure 5), meaning that men who preferred to be relatively taller in the relationship also preferred being relatively active in sexual encounters and vice versa. However, the actual relative height difference among partners was not significantly associated with a preferred sex-role (r_s_ = .075; p = .256; N = 230; Figure 5). Moreover, height of the respondent did not correlate with a preferred sex role (r_s_ = −.027; p = .680; N = 230).

## Discussion

In this study, we first examined preferences for relative height among non-heterosexual men. We showed that most men prefer a partner taller than themselves, but not too much taller. Moreover, a man’s own height was positively associated with the preferred relative height difference of partners. Specifically, taller men preferred relatively shorter partners, whereas shorter men preferred relatively taller partners compared to themselves. These results are in line with studies on heterosexual female mate preferences, which show a general preference for taller men, modulated by a woman’s own height [5]. Our results are thus in agreement with Pawlowski [10], who argued that individuals adjust their height preferences according to their own stature, possibly as a way to increase the pool of their potential partners. Male homosexual partner preferences cannot simply be reduced to a heterosexual female pattern of preferences, however, because almost a quarter of our homosexual sample preferred partners shorter than themselves (a pattern more similar to that observed in heterosexual men). Rather, it seems that men with more gender typical traits (here men of taller stature) show more male-like preferences for shorter partners, while shorter men show more female-like preferences for taller partners. Presenting average partner preferences may therefore obscure these distinctive patterns of partner preferences, perhaps reflecting diverse homosexual sub-groups.

In addition to examining preferences for partner height, we also examined how well these preferences aligned with actual relative height differences among partners. Interestingly, we found a discrepancy between what men preferred, and the actual heights of their partners Although tall men preferred to be taller than their partners and short men preferred to be shorter than their partners, in general, men in our sample expressed a preference for smaller partner height differences than they actually experienced; a finding that was most pronounced in tall men. In other words, taller men would prefer a partner that was taller than their actual partner, but not one who was taller than themselves. This is in line with previous research showing a discrepancy between mate choice preferences, and actual mate choice [6], [8], [40], [60]. Preferred and actual partner characteristics may differ for several reasons because mate selection process usually involves various trade-offs. When choosing a romantic/sexual partner, many characteristics have to be taken into account, and it is unlikely that a particular individual will satisfy all of them. Additionally, rivals may prevent the individual from obtaining the most desired partner. Alternatively, a desired partner may not reciprocate the attraction shown toward them by a particular individual. Among sexual minorities, the discrepancy between mate preference and mate choice may be even more pronounced than among heterosexuals because the potential pool of partners is substantially more limited. Furthermore, when preferences are rather uniform in the population, they are unlikely to be met for most individuals. For instance, the fact that half of the men in our sample preferred a taller partner while only one quarter preferred a shorter partner, must inevitably lead to a compromise, and the acceptance of partners of non-preferred heights by some men.

In line with a growing body of research on assortative mating (for a review see Sterbova & Valentova 2012 [61]), one quarter of men from our sample preferred a relationship with a potential partner of the same height, and a similar proportion of men actually were in a relationship in which partners were of similar height. Similarly, over one third of our sample showed a preference for equal roles during a sexual encounter (i.e., they preferred to be neither passive nor active during sexual activities, or they switched roles regularly), which is in agreement with a previous research on Chinese homosexual men [55]. Moreover, almost half of our sample reported homogamy (i.e., positive assortment) in terms of both preferred and actual relationship dominance. This suggests that a significant proportion of non-heterosexual men in our sample showed preferences for self-similarity in the studied characteristics. This pattern has been repeatedly documented in studies of heterosexual mate preferences and mate-choice, showing that couples resemble each other in basic demographic, personality, and physical characteristics [61].

A final aim of our study was to examine the interplay between height preferences and preferences for hierarchical position within a dyad, which we assessed by both the preferred role during sexual intercourse (‘top’ versus ‘bottom’) and by the preferred dominance role within the relationship (dominant versus submissive behaviour toward a partner). Men who preferred to be ‘top’ in the dyad preferred shorter partners, whereas men who preferred to be ‘bottom’ preferred taller partners. It thus seems that preferences for height are reflected in preferences for hierarchical position within the dyad. This is in agreement with an earlier study showing that homosexual men preferring to be ‘tops’ prefer feminine male faces, while ‘bottoms’ preferred more masculine male faces [52]. This pattern was also observed with respect to preferred relationship dominance status: those men that preferred to be more dominant towards their partner preferred relatively shorter partners, whereas those men that preferred to be more submissive towards their partner preferred relatively taller partners.

A hypothesized reason for why taller male height is preferred by heterosexual women, is that human height is positively associated with measures of social status, such as education and socioeconomic position [34], [35]. Indeed, heterosexual women display stronger preferences for both height and socioeconomic status compared to heterosexual men. Homosexual men may similarly prefer taller men because of the association between height and social status, but very few studies have addressed the preference for socioeconomic status in homosexual individuals. A study based on a US sample, comparing the mating psychology of homosexual and heterosexual individuals, showed that homosexual men, as well as heterosexual men and homosexual women, show less interest in the social status of their partner than do heterosexual women [42]. A more recent study with a sample of Dutch men and women [62] showed that homosexual men, as well as heterosexual men and women put stronger emphasis on socioeconomic status as a partner characteristic than homosexual women. Preferences for socioeconomic status may thus be dependent on local cultures and this issue should be addressed in future studies.

A potential limitation of our study is that participants were mostly recruited via email lists (e.g., [49]), or through advertisements posted on online social networks. Thus, only men who frequently use the internet or email were able to participate in the study, which might potentially bias the results, if homosexual men sampled via social networks differ from homosexual men recruited via lonely-heart advertisements, or via gay bars or at gay parades. With this caveat in mind, this method did, however, enable us to recruit a relatively large sample of non-heterosexual men. Further, our sample was composed of rather non-heterosexual men, thus of both bisexual and predominantly and exclusively homosexual men. Although results of analyzes ran without bisexuals and only with exclusive homosexuals yielded nearly identical results as with the whole sample, more research is needed to investigate specificities of partner preferences of homosexual and bisexual individuals. Also, the relationship between sexual or relationship dominance and height preferences has not been studied in heterosexual individuals, and future studies should address this point also in heterosexual men and women.

As the study was conducted online we were not able to measure actual body height of the participants and instead relied on self-report. In general, self-report is prone to various biases and this might include reliable assessment of height. For example, men who value being taller than their partner might also exaggerate their own height, whereas men who do not value being taller might report more realistic values. There is indeed some evidence that shorter men tend to overestimate their height [65].

Another limitation of our study was that we asked about partner preferences in general terms, rather than specifying whether it concerned short-term or long-term relationships. Preferences may be dependent on such mating-contexts. For example, heterosexual women tend to prefer more masculine traits in short-term male partners, whereas they prefer more feminine features in a long-term relationship context [63], [64]. Whether context dependent fluctuations in mate preferences would also appear in homosexual men is currently unknown, and would be an interesting avenue for future studies.

Despite these limitations, this is one of the first studies on partner preferences in a large sample of non-heterosexual men. We have shown that, although a large proportion of non-heterosexual men prefer to be in an equal relationship with respect to relative height and dominance (although this did not hold for preferred sexual role), preferences for relative height among partners are strongly related to preferences for dyadic dominance in both sexual activities and in terms of the relationship dynamic more generally. We have furthermore shown that mate preferences in homosexual men cannot be simply reduced to gender stereotypes (in this case, gender atypical - resembling preferences of heterosexual females, which would here equate to a preference for taller partners), given that a substantial portion of homosexual men also preferred shorter partners. Moreover, these partner preferences are condition-dependent, influenced by many factors, including own height, preferred dominance hierarchy within a relationship and preferences for active versus passive roles during sexual intercourse.
